# Exploring Nanosilver-Coated Hollow Fiber Microfiltration to Mitigate Biofouling for High Loading Membrane Bioreactor

**DOI:** 10.3390/molecules24122345

**Published:** 2019-06-25

**Authors:** Huy Quang Le, Alieu Sowe, Shiao-Shing Chen, Chinh Cong Duong, Saikat Sinha Ray, Thanh Ngoc-Dan Cao, Nguyen Cong Nguyen

**Affiliations:** 1 Institute of Environmental Engineering and Management, National Taipei University of Technology, No.1, Sec. 3, Zhongxiao E. Rd. Taipei 10608, Taiwan; huylq@dlu.edu.vn (H.Q.L.); alieusowe91@yahoo.com (A.S.); duongchinh73@gmail.com (C.C.D.); ssinharay6@gmail.com (S.S.R.); caothanh201@gmail.com (T.N.-D.C.); 2 Faculty of Environment and Natural Resources, Dalat University, 01 Phu Dong Thien Vuong Street, Da Lat City 66000, Vietnam; nguyennc@dlu.edu.vn; 3 Southern Institute of Water Resources Research, 658 Vo Van Kiet Street, District 5, Ho Chi Minh City 700000, Vietnam; 4 Nguyen Tat Thanh University, 300A Nguyen Tat Thanh Street, District 4, Ho Chi Minh City 700000, Vietnam

**Keywords:** microfiltration membrane bioreactor, silver nanoparticles, membrane modification, membrane biofouling mitigation/control

## Abstract

For the first time, a nanosilver-coated hollow fiber microfiltration (MF) was fabricated by a simple chemical reduction method, then tested for membrane biofouling mitigation study under extreme high mixed liquor suspended solid (MLSS) concentration for long term. This study presents a simple and novel technique to modify a commercially available MF membrane using silver nanoparticles (AgNPs) followed by an investigation of mitigating membrane biofouling potentials using this modified membrane to compare with an unmodified membrane for 60-day operation period. The modified membranes showed that AgNPs was attached to the MF-membrane successfully with a high density of 119.85 ± 5.42 mg/m^2^. After long-term testing of 60 days in membrane bioreactor with a MLSS concentration of 11,000 mg/L, specific flux of the AgNPs coated MF (AgNPs-MF) decreased 59.7%, while the specific flux of the unmodified membrane dropped 81.8%, resulted from the increase of transmembrane vacuum pressure for the AgNPs-MF was lower than that of the unmodified one. The resistance-in-series model was used to calculate the resistance coefficients of membrane modules, and the result showed that the cake layer resistance coefficient of the unmodified membrane was 2.7 times higher than that of the AgNPs-MF after the 60-day operation, confirming that AgNPs displayed great antimicrobial properties to mitigate membrane biofouling under such high MLSS.

## 1. Introduction

Since Yamamoto and his colleague proposed the idea of submerging membranes in the bioreactor in 1989, the membrane bioreactor (MBR) has become a technology that competes with conventional activated sludge process. This process has several advantages, such as high organic loading rate, due to the high mix liquor suspended solid concentration (MLSS) in the bioreactor, lower excess sludge production caused by higher sludge retention time (SRT), lower physical footprint owing to shorter hydraulic retention time (HRT), and excellent effluent water quality [1,2,3]. In spite of its reputation as a secure and high operational reliability technology, membrane fouling is still a major problem that hinders the widespread and large-scale applications [4]. Many studies focused on antifouling strategies to mitigate membrane fouling, such as changing operation conditions by changing the HRT, food to microorganism ratio, air sparging, or membrane filtration cycle [5,6,7].

Membrane surface modification or antifouling-membranes fabrication is another choice for mitigating membrane biofouling, and silver nanoparticles (AgNPs) is a promising option. Previous studies tried to apply AgNPs for mitigating membrane biofouling with different membrane fabrication/modification techniques, as shown in Table 1. In brief, there were two ways to deposit/coat AgNPs on the membrane surface: (1) Fabrication with a mixture of AgNPs in the membrane manufacturing process (2) modification available membrane with coating technique. From previous studies, it could be confirmed that the ability of AgNPs for antibacterial potential and membrane anti-biofouling are noticeable. In various membrane materials, such as polyethersulphone (PES), polysulphone (PS), polyamide (PA), polyvinylidene fluoride (PVDF), polysaccharide, and inorganic structures, silver nanoparticles have been successfully introduced. AgNPs was also used for changing the membrane surface properties in respect of hydrophilicity, which has been thought to improve membrane performance [8]. These techniques have been applied for RO (reverse osmosis), FO (forward osmosis), UF (ultrafiltration), NF (nanofiltration) and MF (microfiltration); however, it was only used for desalination, potable water treatment or testing with bacterial strain, and there have been no studies using AgNPs coated MF membrane to reduce membrane fouling in MBR under high MLSS concentration, where microorganisms have high activities and densities. Therefore, the aim of this study is to modify a commercially available MF membrane with AgNPs to control fouling in the MBR system, with specific focuses on the following aspects: (1) Conduct a simple and novel method for coating the AgNPs on the available MF membrane and characterization of the modified membrane; (2) compare the AgNPs-MBR with a traditional MF-MBR under high MLSS with a long term operation; and (3) evaluate biofouling mitigation by the AgNPs-MBR.

## 2. Results and Discussion

### 2.1. Characterization of the Modified AgNPs-MF Membrane

**Figure 1:**
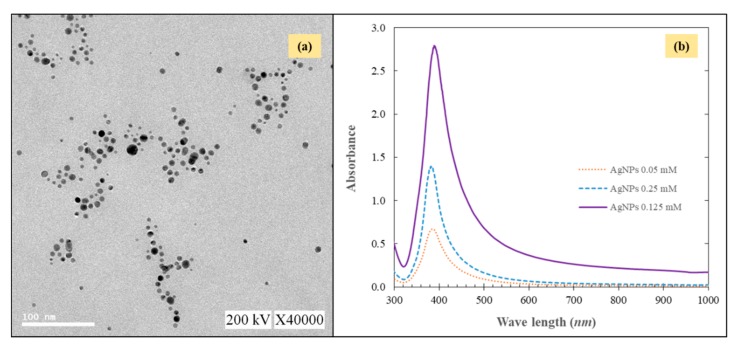
TEM image (**a**) and (**b**) UV-vis absorption spectrum of the AgNPs.

TEM and the UV-vis spectrometry (Figure 1) were used to analyze the synthesized AgNPs which were synthesized via the reduction of silver nitrate by sodium borohydride to make sure that reaction could produce AgNPs. The results from the TEM image showed that the AgNPs were spherical and well dispersed with particle size distribution in the range of 1–100 nm. UV-vis demonstrated that the spectra of the AgNPs suspension revealed a well-defined plasmon band about 380 nm and the peak intensity increased proportionately as the AgNPs concentration increases.

**Figure 2:**
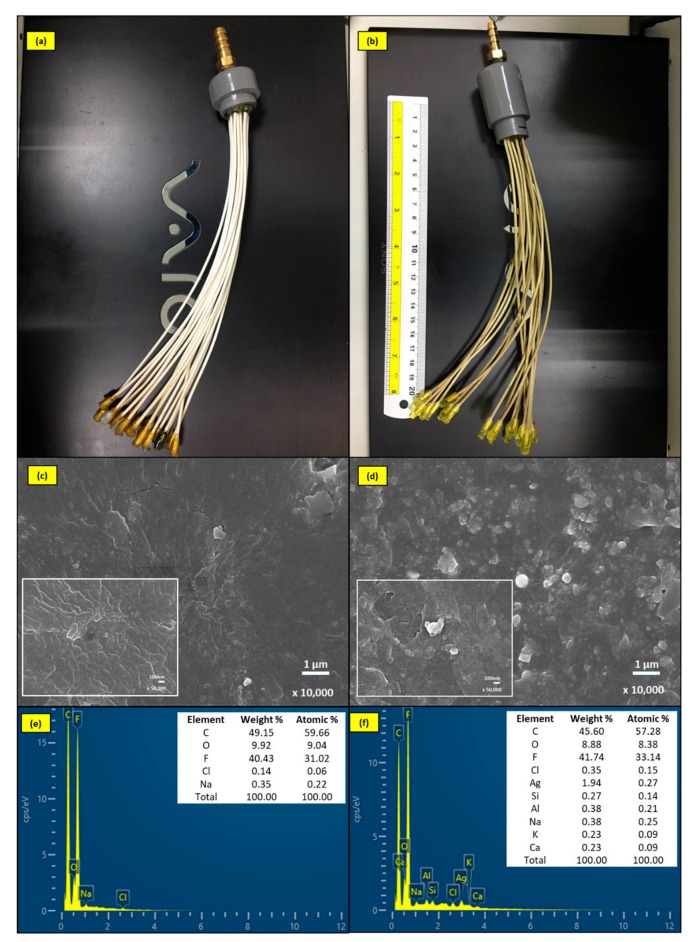
Pictorial representation of (**a**) the unmodified membrane and (**b**) the AgNPs-MF membrane; SEM image of (**c**) unmodified membrane, (**d**) AgNPs-MF membrane and EDS spectra of (**e**) unmodified membrane and (**f**) AgNPs-MF membrane.

SEM/EDS was used to study the morphological properties of the modified and unmodified MF-membranes throughout the experiments, as demonstrated in Figure 2. After coated with AgNPs, the reaction between the membrane and the synthesized AgNPs suspension turned the membrane to yellowish color, indicating the successful coating of AgNPs on the membrane surface (Figure 2a,b) [22]. The SEM analysis showed that there were no AgNPs identified on the surface of the original unmodified membrane, since it was smooth and homogeneous (Figure 2c), and the EDS micrograph indicates the major compositions of the original membrane, such as C, F, and O elements (Figure 2e). As a result of the modification process, the SEM images showed that the surface of the modified membrane was heterogeneous with many spots. These spots with the size from 0.5 μm to smaller than 100 nm located on the membrane (Figure 2d) and it has been revealed by the EDS analysis, confirming the coating of AgNPs on the membrane surface. It was demonstrated that the modification process had changed the membrane surface uniformity which was composed of C, F, O and Ag elements, besides that, a very limited number of other elements are also present which has been indicated in Figure 2f. The ICP analysis was used to determine the quantity of silver attached on the membrane, and the result showed that the quantity of AgNPs detected on the membrane was 119.85 ± 5.42 mg/m^2^ per unit area of the membrane.

### 2.2. Performance of Membrane Bioreactor

**Figure 3:**
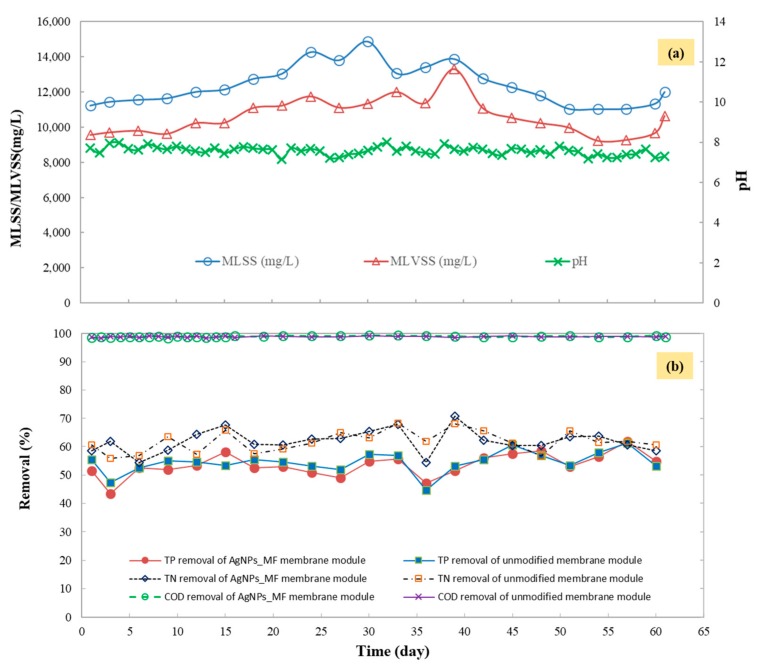
MF-MBR performance in terms of: (**a**) Operation condition, (**b**) removal of Chemical Oxygen Demand (COD), total nitrogen (TN) and TP of unmodified membrane module and the AgNPs-MF membrane module. The dissolved oxygen and temperature of the bioreactor were maintained in the range of 3−4 MgO_2_/L and 25–30 °C, respectively.

Figure 3 shows the performance of bioreactor in terms of operation condition, removal of COD, TN and TP. The MBR was maintained in good conditions from the preliminary phase to the end of the experimental phase to ensure a suitable environment for bacterial growth. The results showed that there is no significant difference between the effluent water quality with the two membrane modules in operation mode (the AgNPs-MF membrane and the unmodified), since the removal percentages of COD and nutrient for this two membrane module were comparable (Figure 3b). The effluent pH varied between 7.0 to 8.0, and the COD removals were greater than 98%, the average removals of TN and TP were 61.4% and 53.5% respectively, while the removals of NH_4_^+^-N were both around 99%. These results mean no negative effect for the AgNPs coated MF to the treatment efficiency and agree with the previous study in which they investigated the effect of AgNPs on activated sludge bacteria when they fed the MBR together with AgNPs concentration of 0.10 mg Ag/L [14].

### 2.3. Membrane Fouling Analysis

#### 2.3.1. Permeate Flux Variation

**Figure 4:**
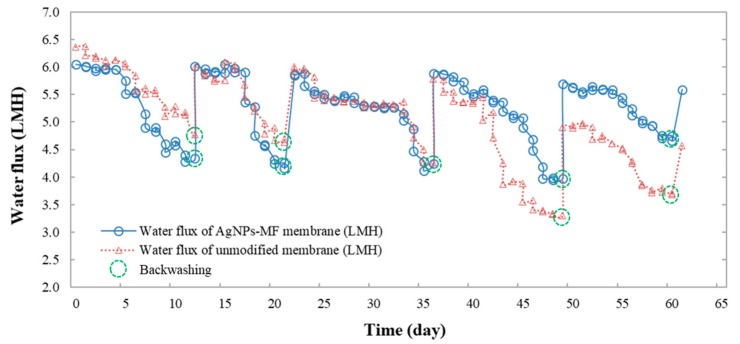
The comparative study of permeate flux of the unmodified membrane module and the AgNPs-MF membrane module over the 60-day operation period.

**Figure 5:**
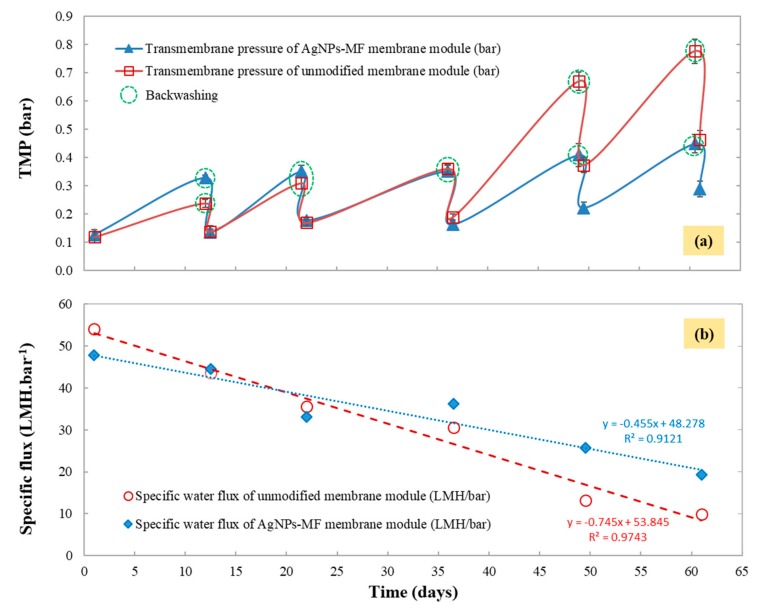
The variation of (**a**) transmembrane pressure (TMP) and (**b**) specific flux variation for the unmodified membrane module and the AgNPs-MF membrane module.

The membrane performance was assessed in terms of water filtration to closely monitor the permeate flux variation throughout the experiment, as shown in Figure 4. After the modification, permeate flux of the AgNPs-MF membrane was found to be 5.6 L/m^2^ h and lower than that of the unmodified MF membrane (6.35 L/m^2^ h). The transmembrane pressure (TMP) of the AgNPs-MF membrane was 0.126 bar, and was higher than the TMP of the unmodified MF membrane (0.117 bar), while the specific flux of unmodified MF membrane was 54.3 L/m^2^ h.bar and higher than the specific flux of AgNPs-MF membrane (48.0 L/m^2^ h bar) (Figure 5). As a result, the membrane resistance of the AgNPs-MF membrane was k_m_ = 8.46 × 10^12^ (m^−1^) and higher than the membrane resistance of unmodified MF membrane (k_m_ = 7.47 × 10^12^ (m^−1^)) (Table 2). These values illustrated that modification had affected the permeate flux of the MF membrane. During the 60-day long term operation, the permeate flux of the unmodified membrane was slightly higher than that of the AgNPs-MF membrane from the first 5 days, after that the permeate flux declined about 25% and 28% from the initial flux of the unmodified and AgNPs-MF membranes, respectively, then a backwashing was done on the 12th days. This cycles were repeated with the backwashing and showed again at the day of 22nd, 36th, 49th and 60th. At the 60th day of continuous operation, the permeate flux for the unmodified membrane decline dramatically from 6.35 L/m^2^ h to 3.7 L/m^2^ h, approximately 41.7% from the original permeate flux even after the physical cleaning, which was a signal for biofouling occurrence. Meanwhile, the AgNPs-MF membrane sustained a slightly stable permeate flux with a decline to 5.6 L/m^2^ h (after backwashing) from the initial permeate flux of 6.0 L/m^2^ h, which is only for 6.7% decline. The biomass growth and the presence of soluble microbial products (SMPs) and extracellular polymeric substances (EPS) can be accounted for the fast fouling rate of the unmodified membrane compared to the AgNPs-MF membrane. 

#### 2.3.2. Transmembrane Pressure Variation Test

When a decline in permeate flux was observed during the experiment, a backwash was performed, and the pressure before and after backwash was measured and recorded. TMP variation test for the modified and the AgNPs-MF membrane modules are given in Figure 5a. A significant decline in permeate flux was firstly observed at the 12th day of the experiment, and a backwash was performed to improve the permeate flux for both membranes. TMP of the AgNPs-MF membrane module was slightly higher than that of the unmodified one in the early days, due to the presence of the AgNPs. At the 22nd, 36th, 49th and the 60th day of continuous operation, TMP of both membranes was measured. During the 60-day of operation, TMP of the unmodified membrane was increased drastically from 0.117 bar to approximately 0.462 bar, while TMP of AgNPs-MF membrane increased from 0.126 bar to 0.289 bar, accounting for 56.22% increase of TMP for the AgNPs-MF membrane and 74.54% increase for the unmodified membrane. The sudden increase of TMP for the unmodified can be attributed to the pore blocking of the membrane, due to the attachment of EPS, SMP, and microbial flocs on the membrane surface, leading to severe flux decline and a drastic increase in TMP.

The specific flux declines in the MFs were investigated by perceiving the membrane fouling as a dynamic process and taking into account the respective TMPs. According to the results of the specific flux in Figure 5b, the initial specific flux for the unmodified and the AgNPs-MF membrane was 54.3 L/m^2^ h.bar and 48.0 L/m^2^ h.bar, and declined to 9.9 and 19.34 L/m^2^ h.bar respectively after 60-day of operation, accounting for the specific flux declined of 59.7% for the AgNPs-MF membrane and 81.8% for the unmodified membrane. Even water backwash cannot recover the specific flux to the original point. Consequently, a linear model was established to distinguish and assess the fouling rate of both membranes for decline rate studies in Figure 5b to establish a fouling pattern for both the unmodified and AgNPs-MF membranes. According to the linear model analysis, the membrane flux decline rate for the unmodified membrane was higher as compared to the AgNPs-MF membrane as the slope of the trendline were 0.745 and 0.445, respectively, indicating faster biofouling rate of the unmodified as compared to the AgNPs-MF membrane.

#### 2.3.3. Resistance Model Analysis

The resistance-in-series model was applied to obtain the membrane resistance coefficients k_m_(m^−1^), the hydraulically reversible fouling resistance coefficients k_h_ (m^−1^) and the cake layer resistance coefficients k_c_ (m^−1^) of the two membrane modules in operation mode, as shown in Table 2. From starting, k_m_ of the AgNPs-MF membrane module was 8.46 × 10^12^ (m^−1^) and higher than k_m_ of the unmodified MF membrane module (7.47 × 10^12^ (m^−1^)). k_h_ of the AgNPS-MF at the day of 12th was higher than k_h_ of the unmodified membrane module, illustrating that the modification affected the permeate flux of the MF membrane. For the first 36 days in operation, k_h_ of the AgNPS-MF membrane module was almost stable, while k_h_ of the unmodified membrane module was increased nearly twice times from day 22nd to day 36th. As the same time, k_c_ of the unmodified membrane module was increased rapidly and got the value of 2.1 times higher than k_c_ of the AgNPs-MF membrane module at day 36th. These results may be caused by higher permeate flux of the unmodified membrane module. From day 36th to day 49th, k_h_ and k_c_ of both membrane modules increased faster, especially with the unmodified module, while permeating flux of the unmodified membrane module has dropped quickly. It might be caused by high MLSS concentration in the bioreactor (Figure 3), and there was enough time for chemical fouling and biofouling occurred on the membrane surface, due to long contact time. As a result, after day 49th, k_c_ of both membrane modules increased, since there was some cake layer formed on the membrane surface, while the k_h_ of these two membrane modules has declined because MLSS in the bioreactor was decreased. Overall, the total resistance of the unmodified membrane module increased quickly from the lower to the higher values, from 10% lower to 2.2 times higher, in comparison with these values of the AgNPs-MF membrane module. Moreover, k_c_ of the unmodified membrane module was always higher than k_c_ of AgNPs-MF membrane module, since day 22, due to the cake layer increased adhesion of the activated sludge under vacuum pressure, and higher values of k_h_ came together with higher k_c_ values, because of the higher absorption area produced by the cake layer. The results demonstrated that the unmodified membrane module possessed more fouling than the AgNPs-MF membrane module as the k_c_ of the unmodified membrane module was 2.7 times higher than k_c_ of the AgNPs-MF membrane after the 60-day operation.

#### 2.3.4. Biofouling Analysis by SEM-EDS

**Figure 6:**
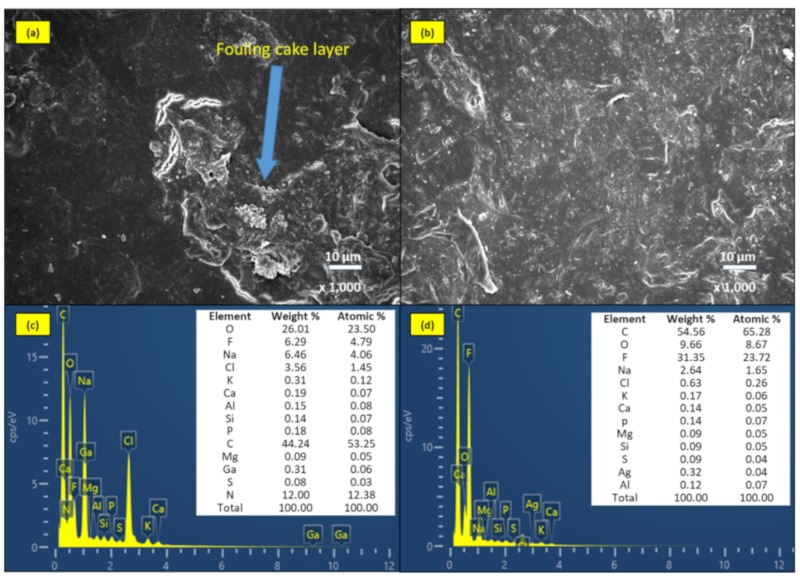
The images of SEM and EDS spectra of (**a**, **c**) unmodified membrane and (**b**, **d**) AgNPs-MF membrane after 60 days in operation mode.

According to the Figure 6 and the resistance-in-series model’s results, the unmodified membrane accumulated more fouling layers on the membrane surface, as compared to the AgNPs coated membrane, which had a smoother membrane surface morphology after long-term filtration test. However, the observations from the SEM analysis showed the thick cake layer like biofouling layer formed onto the unmodified membrane surface, while there was no thick cake layer on the AgNPs-MF membrane surface. Moreover, the resistance-in-series model demonstrated that the unmodified module showed more fouling than the AgNPs-MF membrane, because the cake layer resistance coefficient of the unmodified was 2.7 times higher than that of the AgNPs-MF membrane after 60-day operation. The EDS results also indicated that membrane surfaces were covered with both organics and inorganics as revealed by the elemental composition of the EDS spectra present for both membranes. There were some elements, such as C, N, and P, which indicated the organic compounds like bacteria cells on both membranes, but high weight percentage of N was only detected on the unmodified membrane. Although, EDS results indicated some inorganic elements on the membrane with the membrane that employed in the bioreactor with high MLSS concentration, the cake layer that was shown in the SEM image was referred to as biofouling. Moreover, towards the end of the experiment, the permeate flux, and TMP of the unmodified membrane drastically dropped (even the backwashing), due to the formation of biofilm and biopolymers on the membrane surface which consequently blocked the membrane pore space, resulting in biofouling of the membrane. This phenomenon was different from the AgNPs coated membrane, which showed less reduction in permeate flux and TMP. This result agreed with other studies that AgNPs coating on the membrane could mitigate biofouling (Table 1). Membrane biofouling in membrane bioreactor was caused by bacteria cell and its product, colloids and chemical solutes. These ingredients accumulated onto the membrane structure, clogging the pores, covering the membrane surface and resulting in membrane permeability decline and cake layer formation, as seen in Figure 5 and Figure 6. These biofoulants not only decreased the membrane permeate flux but also created a favorable environment for bacteria to colonize and develop on the membrane. Since the ability of AgNPs for antibacterial, the bacterial cell cannot attach to the membrane, therefore it cannot multiply for attached cell cluster to become bio-cake on the membrane surface or inside the membrane pores, as demonstrated in Figure 6. During the operation mode, the bacterial cell might be pushed on the membrane surface or into the membrane pores by TMP or other operation conditions. However, under the effect of the AgNPs, it cannot form on the membrane and easy to be washed out by the backwashing process, the water flow on the membrane surface or the air bubble of the aeration in the bioreactor.

**Figure 7:**
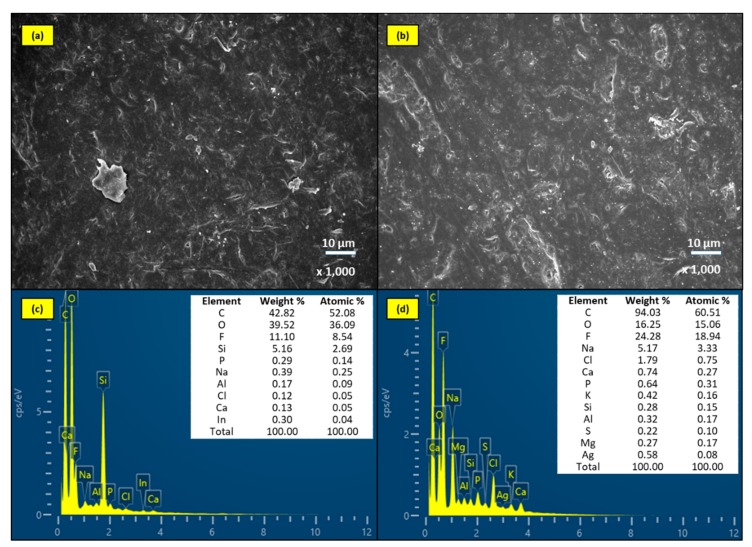
The images of SEM and EDS spectra of (**a**, **c**) unmodified membrane and (**b**, **d**) AgNPs-MF membrane after 60 days in submerged mode.

SEM and EDS spectra of the unmodified membrane and AgNPs-MF membrane modules in the submerged mode were shown in Figure 7 which illustrated that no significant change was observed on these membrane surfaces after 60-days of dipping in the bioreactor. There was no noticeable fouling layer on these membranes, since they have smoother surfaces in comparison with the two membrane modules in operation mode. However, the EDS spectra pointed out that there were some organic and inorganic elements on that. The presence of inorganics is due to slight scale depositions because of polarization, while the organic should be referred to bacteria attached growth on the membrane, since the bioreactor was operation with a high concentration of MLSS. For AgNPs-MF membrane in submerged mode, the EDS spectra present there was Ag element, therefore it was illustrated for long lasting of attached AgNPs on the membrane. The inorganic elements were found on the AgNPs-MF membrane were more diversity than that on the unmodified membrane might be the result of attached AgNPs have changed the charge of the membrane surface, thereby promoting precipitation of inorganic substances. Biofouling was also the reasons for increasing the absorption of inorganic elements to the membrane surface. The smoother surfaces of the submerged mode membrane modules in comparison with operation mode membrane modules’ surface demonstrated that the membrane in the MF-MBR was gradually fouled under the filtration process, due to vacuum pressure, facilitating the bacteria and other bulk matter attached and grown on the membrane surface and inside the membrane structure.

#### 2.3.5. Stability of AgNPs Coated Membrane and Silver Leaching Analysis

**Figure 8:**
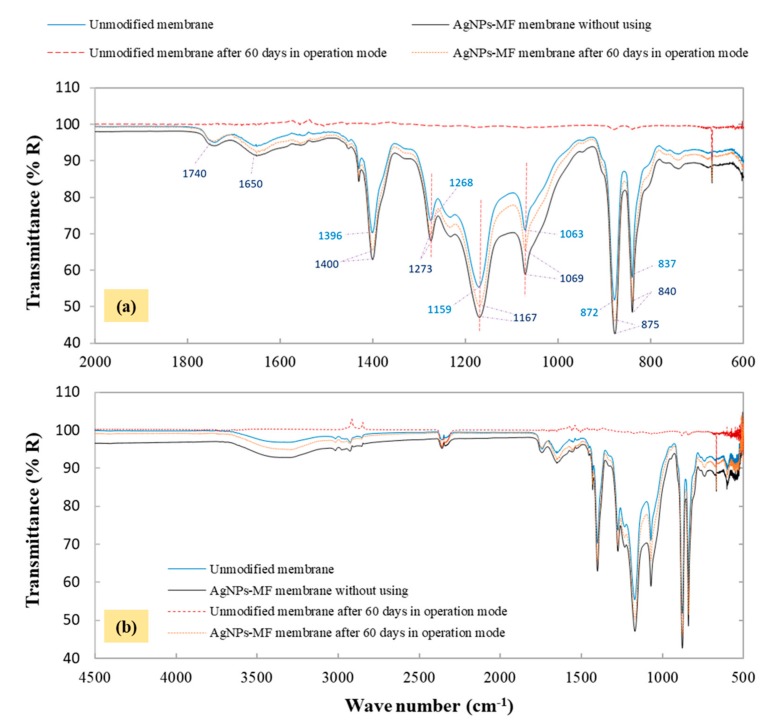
FTIR spectra of the membranes analyzed before and after the experiment in the wavenumber range of (**a**) 2000 to 600 cm^−1^ and (**b**) 4500 to 500 cm^−1^.

The nanoparticles abundance before and after the experiment were measured as a function of the silver concentration on the membrane using ICP analysis. The ICP analysis showed that the quantity of silver detected on the membrane was 119.85 ± 5.42 mg/m^2^ per unit area of the membrane in the beginning, and dropped to 117.09 ± 4.68 mg/m^2^ after the experiment. This result showed that no significant leaching occurred from the AgNPs-MF membrane during the experimental period. Thus, the stability of the AgNPs on the membrane suggests that the antibacterial effects of AgNPs could exist for a long time. FTIR analysis had been done for the membranes before and after the experiment (Figure 8) and the absorption bands for original membranes were presented at 1396 cm^−1^, 1268 cm^−1^, 1159 cm^−1^, 1063 cm^−1^, 872 cm^−1^ and 837 cm^−1^, those were referred to –CH_2_- in PVDF chain, in-plane C-H blending, β-crystal of PVDF (CF_2_ stretching), α-phase of PVDF, an amorphous structure of PVDF (CF_2_ stretching) and β-phase of PVDF (CH_2_ and CF_2_), respectively [23,24]. After the modification, FTIR spectra of the membrane with AgNPs also had the same absorption band, but these reduced transmittance and the peak shifts to lower wavenumber as 1400 cm^−1^, 1273 cm^−1^, 1167 cm^−1^, 1069 cm^−1^, 875 cm^−1^, and 840 cm^−1^, respectively (Figure 8a). This phenomenon could be the result of the metal, such as silver coordinated with atoms like halogen, hydro in the structure of the membrane and produces the characteristic infrared band and might be the mechanism that AgNPs attached to the membrane. Some previous studies that described the same phenomenon when modified PVDF membrane with silver or other metal [23,25,26]. On the unmodified membrane in the operation mode, a new band stretching was detected at 2930 cm^−1^, attributed to C-H_2_ group from the organic compounds [24], indicating the growth of organic compounds. Such bands were not found on the AgNPs modified membrane. In addition, bands formed at 3300 cm^−1^ was also, due to N-H stretching [24,27] because of the organic matter attached on the membrane surface. The formation of organic compounds on the surface of the unmodified as indicated by the C-H_2_ group could also be attributed to the biomass growth on the membrane, causing severe biofouling of the membrane.

## 3. Material and Methods

### 3.1. Nanosilver Coated Membrane Fabrication

In this work, specific membrane modules were prepared from the MF membrane strings which are hollow fibers, made of sterapore polyethylene material with a pore size of 0.1 μm obtained from Mitsubishi Rayon co., Ltd. Japan, and each membrane module has an active membrane area of 19.1 cm^2^. In total, four membrane modules were prepared, in which two of the membrane modules were coated with AgNPs, and the other two were unmodified to differentiate the effects of AgNPs for biofouling mitigation studies. Two of these membrane modules (one unmodified and the other coated with AgNPs) were filtered in the operation mode to assess the membrane biofouling during the 60-day experimental period. Simultaneously, the other two membranes were not operated, but submerged into, the bioreactor (submerged mode) to analyze and compare the effects of vacuum pressure, permeate flux on biofouling, and the nature of attached growth of bacteria on the membrane material during the long term experimental process.

To make sure that the chemical reaction which has been used to modify the membrane could produce AgNPs, the chemical reaction was done without membrane by adding dropwise 10 mL solution of silver nitrate, 0.005 M AgNO_3_ into 30 mL sodium borohydride solution, 0.01 M NaBH_4_ at 4 °C which was slowly mixed at 20 rpm. After that, characterization of the AgNPs-MF was performed to confirm the successful synthesis. At first, the MF-membrane strings were cut and immersed into 0.05% HCl for 1 h, after that the membrane was then taken out and washed with DI water followed by continuous drying for 8 h at room temperature. The membrane was dipped in a prepared solution of silver nitrate, 0.005 M AgNO_3_, for 1 h after drying, and then taken out from the AgNO_3_ solution to dip into 4 °C sodium borohydride solution, 0.01 M NaBH_4_ for another 1 h. The completed modified membrane was taken out of the beaker with NaBH_4_ solution and washed with DI water to remove impurities and then dried overnight at room temperature to ensure that the AgNPs was properly attached stably to the membrane. Finally, the resulted membrane was tested for characterization using Scanning Electron Microscope (SEM), Energy Dispersive Spectroscopy/X-ray (EDX), transmission electron microscopy (TEM) and Fourier transform infrared (FTIR) to confirm AgNPs attachment on the membrane surface before and after the experiments. SEM (JEOL, JSM-5900, Tokyo, Japan) and EDX (Philips, XL-30 W/TMP, Eindhoven, Holland) were used to thoroughly analyze the membrane surface morphologies, TEM (Hitachi, H-7100, Tokyo, Japan) provided the image transmitted through the AgNPs, UV-vis spectrophotometer (JASCO V-550, Tokyo, Japan) was used to characterize the synthesized AgNPs, and FTIR spectra (Waltham, MA, USA) was to identify the functional group of the modified membrane.

Concentration and stability of the synthesized AgNPs on the membrane surface were analyzed after the attachment to the membrane by a chemical reduction method. The quantity of AgNPs attached to the membrane surface was determined by inductively coupled plasma optical emission spectrometry (ICP-OES, Perkin Elmer Optima 8000, Perkin Elmer, Inc., Waltham, MA, USA). In this analysis, a piece of the membrane with a known area was cut and dissolved in high concentration HNO_3_ (32.5%), and the resulted solution was centrifuged and analyzed by the ICP. Stability of the AgNPs was also determined at the end of the experimental phase through this silver leaching analysis.

### 3.2. MF-MBR Experimental Setup

**Figure 9:**
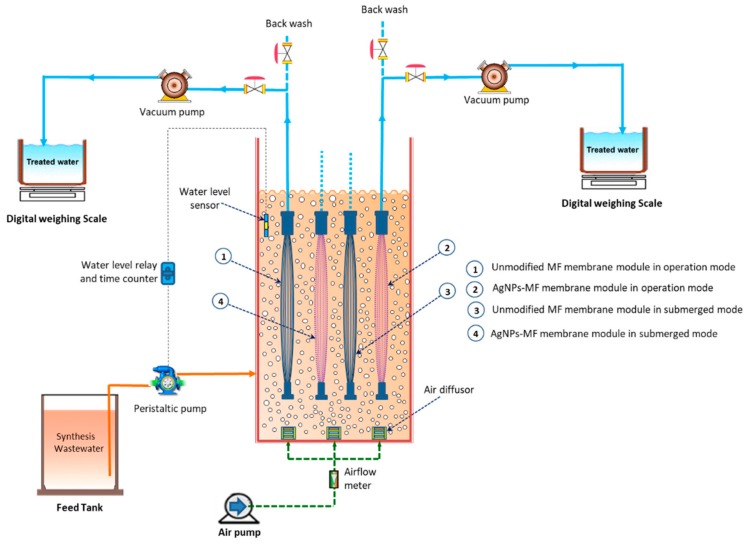
Schematic of the experimental setup for lab-scale MF-MBR system.

Figure 9 provides a pictorial presentation of the lab setup of the MF-MBR system. An MF-MBR in column shape with the effective volume of 6 litters was conducted in a lab setup. The results were recorded for evaluating the long-term performance of the AgNPs in mitigating membrane biofouling. The aerobic activated sludge was collected from Danshui wastewater treatment plant, Taipei, Taiwan and fed in the bioreactor for three weeks before using in the experiments. The MLSS was set at 11,000 mg MLSS/L, while the mixed liquor volatile suspended solids (MLVSS) was 9000 mg MLVSS/L. Chemical Oxygen Demand (COD) around 1100 mg/L composed of glucose (C_6_H_12_O_6_), ammonium chloride (NH_4_Cl) and potassium dihydrogen phosphate (KH_2_PO_4_) were used as the feed solution to maintain the ratio of COD: N: P is 100:5:1, respectively. From the beginning, the F/M ratio of the system was 0.1 day^−1^ with an organic loading rate of 1.1 kg COD/m^3^ day, while the SRT of the bioreactor was maintained at 50 days. One peristaltic pump maintained at a velocity of 6 L/day was used for feeding, and two vacuum pumps were employed for evacuating the permeate from the two membrane modules which were in operation mode. The two vacuum pumps were set at 3 L/day from the beginning, and the transmembrane pressure was automatically balanced and recorded by a manometer. As the permeate flux of the two membrane modules may fluctuate during the operation time, the feed pump working time was controlled to keep the water level in the bioreactor stable by a water level relay that was connected with a water level sensor in the bioreactor. A time counter was used to record working time of the feed pump. HRT of the bioreactor was changed from 1.0–1.7 day, due to permeating fluxes of two operation membrane modules that were changing over the operation period.

An air pump provided the air volume of 5 L/min through the air diffuser at the bottom of the bioreactor to keep the dissolved oxygen in the bioreactor between 3−4 mg/L. The bioreactor temperature was maintained between 25 to 30 °C, and NaHCO_3_ was used as the buffer for alkalinity adjustment to maintain a suitable microbial growth.

The above MF-MBR system consists of four membrane modules, in which two modules were coated with AgNPs, while the other two were left unmodified. The two MF membrane modules (one was coated with AgNPs, and the other was unmodified) were in the operation mode, and the other two were in submerged mode. In the system, two of the membrane modules in operation mode (number 1 and 2 in Figure 9) were tested for water filtration performance and TMP variation to assess the biofouling potential for each module in the operation mode during the long-term experimental period. However, the other two membranes in the submerged mode (number 3 and 4 in Figure 9) were submerged in the bioreactor throughout the experimental period to assess the rate of biofouling susceptibility compared to those membranes in the operation mode Table 3 described the four membrane module characteristics and operation mode. Two effluent tanks were used to collect the effluent, which was pumped out through the two membrane modules in the operation mode by two vacuum pumps, respectively. Transmembrane pressure of each membrane module was measured by a manometer and permeate flux of each membrane module was calculated from the volume change, which was measured before and after by a digital weighing balance. During the time of operation, when the permeate flux through the membrane modules was reduced 30%, the two membrane modules in operation mode were taken out to backwash by distilled water with a flow rate of 80 mL/min for 10 min to reduce physical fouling of the membranes. After the backwashing, the membrane modules were put back into the bioreactor, and TMP variation was tested again. During the filtration test at each point before and after backwashing, TMPs for the modified and unmodified MF were recorded again by a manometer.

Removal of COD and nutrient of each working membrane module was calculated using Equation (1):

(1) $$R\left(\%\right)=\left(1-\frac{C_{e}}{C_{0}}\right)\times 100,$$

where R (%), C_0_ (mg/L) and C_e_ (mg/L) are the removal percentage, influent concentration and effluent concentration of the COD/nutrient of the permeate that collected from each membrane module.

Permeate flux, J_w_ (L/m^2^ h; LMH), was calculated in terms of the total volume of the permeate collected using the given Equation (2):

(2) $$J_{w}=\frac{V}{A\Delta t},$$

where V (L) and A (m^2^) are the volumes of the permeate tank over a specific period of time ∆*t* (h) and effective area of the membrane used during the MF-MBR process, respectively. The specific flux of each membrane was calculated using Equation (3):

(3) $$J_{s}=\frac{J_{W}}{\Delta P},$$

where J_s_ is the specific flux of the membrane, J_w_ is the permeate flux, and ΔP is the transmembrane pressure respectively.

### 3.3. Membrane Fouling Analysis

Permeate flux, the specific flux of the membrane variation and TMP variation test were conducted to assess the fouling potentials of the membranes. TMP was tested during the long-term filtration test at each point before and after backwashing. The backwashing was executed to remove the physical fouling on the membrane surface, but the chemical fouling and biofouling were still attached on the membrane surface. Therefore, an increase in the transmembrane vacuum pressure in each case was a signal for membrane fouling, especially biofouling. The percentage increase in transmembrane pressure across each membrane was calculated by the following equation:

(4) $$\Delta P\;\left(\%\right)=\frac{P_{\mathrm{BF}\;}-{\;P}_{\mathrm{AF}}}{P_{\mathrm{BF}}}\;\times 100,$$

where ΔP (%) is the percentage increase in the transmembrane pressure, P_BF_ is the transmembrane pressure before fouling, and P_AF_ is the transmembrane pressure after fouling, respectively. The membrane fouling can be expressed by a resistance-in-series model [28] as Equation (5). The resistance-in-series model applies a resistance value to three main components of membrane fouling in the current study, including the membrane resistance coefficient k_m_ (m^−1^), the hydraulically reversible fouling resistance coefficient k_h_ (m^−1^) and the cake layer resistance coefficient k_c_ (m^−1^) in relation with permeate flux J_w_ (L/m^2^.h), transmembrane pressure ΔP (Pa) and water dynamic viscosity µ (kg/m.s),

(5) $$J_{w}=\frac{\Delta P}{\mu (k_{m}+k_{h}+k_{c})}.$$

From the first day of operation, when the system just started in the bioreactor and k_h_ = k_c_ = 0, the value of k_m_ can be calculated, and k_m_ is a constant for all the experiments. Then the total membrane resistance was k_m_ + k_h_ + k_c_ before the backwashing, and k_h_ = 0 at the point of time immediately after backwashing, so the values of k_h_ and k_c_ could be obtained. Equation (5), together with the experimental data, can be used for calculating the value of k_h_ and k_c_ of each membrane module during the operation time. After the experiment, the four used membrane modules were taken out of the bioreactor and washed with DI water, then a piece of each used membrane was cut and dried with nitrogen gas in 2 h before stored in a desiccator. SEM and EDS were again used to examine the membrane surfaces before and after the experiment for evaluating membrane fouling. FTIR was also used for the fouling analysis to show possible functional groups of the fouled membrane compared to the unmodified membrane.

### 3.4. Analysis of Chemical and Physical Parameters

COD and phosphate were measured by the calorimetric method using spectrophotometer (DR 6000, HACH, Loveland, Colorado, USA). The amount of total nitrogen (TN) was analyzed by the sum of ammonia-nitrogen (NH_3_-N), nitrite-nitrogen (NO_2_^−^-N) and nitrate-nitrogen (NO_3_^−^-N). The temperature, dissolved oxygen, and pH of the bioreactor was measured with a multi-parameter water quality meter (YSI Inc., Yellow Springs, Ohio, USA). MLSS, MLVSS, nitrate and nitrite were measured using Standard methods [29]. The transmembrane vacuum pressure was measured by digital manometers (Koang Yee Enterprise Co., Ltd., Taipei, Taiwan).

## 4. Conclusions

In this study, the modification and characterization of hollow fiber MF membrane based on coated AgNPs for mitigating biofouling in high loading membrane bioreactor were investigated. Main conclusions can be listed as follows:

(1) AgNPs coating membrane was successfully fabricated by a simple novel technique. TEM, SEM, EDS and ICP analysis showed that the membrane was coated by high density AgNPs, and there was no significant leaching observed from the AgNPs-MF membrane during the experimental period. Thus, the stability of the AgNPs on the membrane suggested that the antibacterial effects of AgNPs could exist for a longer time in an aerobic bioreactor under high MLSS condition.
(2) There was no adverse effect of the AgNPs to the MF-MBR treatment efficiency on the bioreactor performance. The effluent water quality from the AgNPs-MF and the unmodified MF were similar after 60 days of operation.
(3) The AgNPs-MF membrane module was effective and had direct impacts on reducing the biofouling of the membrane. The AgNPs-MF membrane was not only having antimicrobial effects, but also prevented the bacteria attachment to the membrane surface, thus, reducing biofilm formation. The current study also showed that the membrane in operation mode caused the membrane clogging and biofouling faster than the membrane just submerged in the bioreactor.

## Figures and Tables

**Table 1:** Summary on membrane fabrication/modification bases on silver nanoparticles (AgNPs) coating technique.

Type of Membrane	Application	Scale	Membrane Fabrication/Modification Techniques	Quantity of AgNPs on Membrane	Duration	Mitigating Membrane Biofouling	References
RO	Seawater desalination	Flat-sheet membrane cell in lab scale	Membrane modification with chemical reduction method (silver nitrate and formaldehyde solutions).	-	20 days	Yes	[9]
UF	Antibacterial and anti-biofouling using bacterial strain	Flat-sheet vacuum filtration cell in lab scale	Membrane fabrication with the wet phase-inversion process (polysulfone with commercial AgNPs)	0.9% (by weight)	24—72 h	Yes	[10]
UF	Antibacterial and anti-biofouling using bacterial strain	disc diffusion method	Membrane modification with biogenic AgNPs	0.1—1.0% (by weight)	12 h	Yes	[11]
NF	Antibacterial and anti-biofouling using bacterial strain	Flat-sheet membrane cell in lab scale	Membrane fabrication (polymeric composite + commercial AgNPs)	0.05—10% (by weight)	24 h	Yes	[12]
NF and FO	Antibacterial and anti-biofouling using bacterial strain	crossflow filtration model in lab scale	Membrane fabrication (fabricated by layer-by-layer (LbL) assembly + commercial AgNPs)	0.22–1.19 (by weight)	48 h	Yes	[13]
UF	Wastewater treatment	Aerobic MBR with submerged hollow fiber UF membrane module in lab scale	No (feeding solution: Synthesis wastewater + 0.10 mg/L AgNPs)	0	65 days	No	[14]
MF	pH and ionic strength study	Dead-end microfiltration system in lab scale	Membrane modification (deposition of AgNPs synthesis by chemical reduction silver nitrate with ammonia solution)	-	-	Didn’t study	[15]
MF	Water disinfection potable water	Dead-end filtration cell in lab scale with raw river water and synthetic feed containing *E. coli*	Membrane modification (AgNPs synthesis by chemical reduction (Silver nitrate, sodium borohydride and ethanol))	0.0139 mg cm^−2^	5 days	Didn’t study	[16]
UF	Antibacterial and anti-biofouling using bacterial strain	Flat sheet bench-scale module	Graphene oxide -nanosilver UF membrane fabrication (Hummers’ method + Turkevich method)	-	24 h	Yes	[17]
UF	Antibacterial and anti-biofouling using wastewater	Flat-sheet vacuum filtration cell in lab scale	Membrane modification with (i) diffusion of silver ions with subsequent reduction, (ii) addition of polyethyleneimine-capped AgNPs, and (iii) thermal-pressure fixation of silver-modified nanofibres	< 0.07–0.92% (by weight)	0.5–8.0 h	Yes	[18]
RO	Water filtration	Flat-sheet membrane cell in lab scale	modification of the spacer y sonochemical deposition (deposition of AgNPs synthesis by chemical reduction silver nitrate with ammonia solution)	9.0% (by weight) on spacer	10 days	Yes	[19]
UF	Antibacterial and anti-biofouling using bacterial strain	Dead-end filtration cell in lab scale	Graphene oxide-nanosilver/PVDF UF membrane fabrication (Hummers’ method + AgNPs synthesis by chemical reduction silver nitrate with ammonia solution)	-	24 h	Yes	[20]
RO	Lake water purification	Flat-sheet membrane cell in lab scale	Membrane fabrication (polyamide-urethane-imide + commercial AgNPs)	-	7 days	Yes	[21]

**Table 2:** Membrane resistance coefficients, hydraulically reversible fouling resistance coefficients, cake layer resistance coefficients and total the resistance of two membrane modules in operation mode calculated by resistance series model.

Time (Day)	AgNPs-MF Membrane Module k_m_ = 8.46 × 10^12^ (m^−1^)			Unmodified MF Membrane Module k_m_ = 7.47 × 10^12^ (m^−1^)		
	k_h_ (m^−1^)	k_c_ (m^−1^)	Total Resistance (m^−1^)	k_h_ (m^−1^)	k_c_ (m^−1^)	Total Resistance (m^−1^)
0	0	0	8.46 × 10^12^	0	0	7.47 × 10^12^
12	2.15 × 10^13^	6.35 × 10^11^	3.06 × 10^13^	1.08 × 10^13^	1.81 × 10^12^	2.01 × 10^13^
22	2.19 × 10^13^	3.76 × 10^12^	3.41 × 10^13^	1.52 × 10^13^	3.87 × 10^12^	2.66 × 10^13^
36	2.22 × 10^13^	2.73 × 10^12^	3.34 × 10^13^	2.09 × 10^13^	5.72 × 10^12^	3.41 × 10^13^
49	2.57 × 10^13^	7.30 × 10^12^	4.15 × 10^13^	5.13 × 10^13^	2.32 × 10^13^	8.20 × 10^13^
60	1.80 × 10^13^	1.24 × 10^13^	3.89 × 10^13^	4.43 × 10^13^	3.34 × 10^13^	8.51 × 10^13^

**Table 3:** MF membrane modules used in the experiments.

Membrane Module	Material/AgNPs Coating	Operation Mode	Shape/Pore Size/Surface Area
1	Polyethylene/unmodified membrane	In operation mode	Hollow fiber/0.1 μm/19.1 cm^2^
2	Polyethylene/AgNPs coated membrane	In operation mode	Hollow fiber/0.1 μm/19.1 cm^2^
3	Polyethylene/unmodified membrane	In submerged mode—dipping in bioreactor	Hollow fiber/0.1 μm/19.1 cm^2^
4	Polyethylene/AgNPs coated membrane	in submerged mode—dipping in bioreactor	Hollow fiber/0.1 μm/19.1 cm^2^
